# Gold-based nanomaterials for the treatment of brain cancer

**DOI:** 10.20892/j.issn.2095-3941.2020.0524

**Published:** 2021-06-15

**Authors:** Li Tu, Zheng Luo, Yun-Long Wu, Shuaidong Huo, Xing-Jie Liang

**Affiliations:** 1 Fujian Provincial Key Laboratory of Innovative Drug Target Research, School of Pharmaceutical Sciences, Xiamen University, Xiamen 361102, China; 2 CAS Center for Excellence in Nanoscience, CAS Key Laboratory for Biomedical Effects of Nanomaterials and Nanosafety, National Center for Nanoscience and Technology, Beijing 100190, China

**Keywords:** Gold-based, nanomaterials, BBB penetration, brain cancer therapy

## Abstract

Brain cancer, also known as intracranial cancer, is one of the most invasive and fatal cancers affecting people of all ages. Despite the great advances in medical technology, improvements in transporting drugs into brain tissue have been limited by the challenge of crossing the blood-brain barrier (BBB). Fortunately, recent endeavors using gold-based nanomaterials (GBNs) have indicated the potential of these materials to cross the BBB. Therefore, GBNs might be an attractive therapeutic strategy against brain cancer. Herein, we aim to present a comprehensive summary of current understanding of the critical effects of the physicochemical properties and surface modifications of GBNs on BBB penetration for applications in brain cancer treatment. Furthermore, the most recent GBNs and their impressive performance in precise bioimaging and efficient inhibition of brain tumors are also summarized, with an emphasis on the mechanism of their effective BBB penetration. Finally, the challenges and future outlook in using GBNs for brain cancer treatment are discussed. We hope that this review will spark researchers’ interest in constructing more powerful nanoplatforms for brain disease treatment.

## Introduction

Brain cancer is one of the most aggressive tumors, and it severely threatens people’s lives, owing to poor treatment efficacy^1^. Currently, clinical surgery, radiotherapy, and chemotherapy can help relieve pain and prolong the survival time of patients with brain cancer. Nevertheless, their curative effects remain unsatisfactory, owing to several inevitable limitations. Typically, physically eradicating brain tumors by surgery is almost impossible, because of the difficulty in distinguishing tumor tissue from normal brain tissue^2^. In radiotherapy, the insufficient sensitivity of brain tumor cells to ionizing radiation in the hypoxic tumor microenvironment results in low therapeutic efficacy^3^. In chemotherapy, which relies on small molecular drugs with tumor inhibition ability^4^, the efficiency of drug delivery is significantly impeded by many factors, e.g., a lack of physiological stability^5^, non-targeting specificity^6^, and the most challenging obstacle: the blood-brain barrier (BBB)^7^.

**Figure 1:**
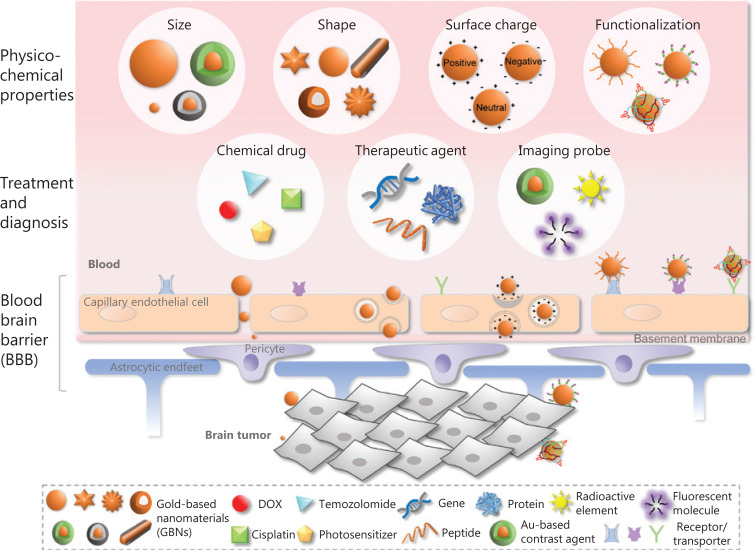
Schematic diagram of GBNs with various physicochemical properties in the delivery of therapeutic and diagnostic agents through the BBB for the treatment of brain cancer.

The BBB, a highly specific dynamic interface located between the blood capillaries and the central nervous system (CNS), is mainly composed of densely packed cerebral capillary endothelial cells (CCECs)^8,9^. In contrast to other capillary endothelial cells, the CCECs in the BBB are encompassed by pericytes and the presynaptic membranes of astrocytes *via* the basilemma (**Figure 1**)^10^. Additionally, the BBB has fewer proteins in the interstitial fluid than in other parts of the body. As a result, the BBB exhibits similar properties to semi-permeable membranes and effectively restricts the free exchange of substances between the blood and brain tissue, thus preventing the entry of harmful exogenous chemicals into the brain tissue^11,12^. However, despite protecting the CNS from damage, the BBB also blocks the penetration of therapeutic agents, thus posing difficulties in treating brain diseases. The BBB restricts the access of more than 98% of small-molecule drugs and nearly 100% of biomolecules^13^. Current methods to cross the BBB include intrathecal injection^14^, osmotic destruction^15^, ultrasound or magnetic interference^16,17^, and nasal administration^18^. Unfortunately, these approaches may require undesired invasive operations and cause substantial trauma, biotoxicity, and potentially damage to the BBB. Therefore, the development of more effective but safe strategies for bypassing the BBB is urgently needed for therapeutic delivery to brain lesions.

The flourishing development of nanotechnology contributes diverse nanomaterials with good biocompatibility for biomedical applications, including micelles^19^, liposomes^20^, and inorganic nanoparticles (NPs)^21^, which have frequently been applied as carriers for therapeutic delivery. Notably, the utilization of NPs for drug delivery provides numerous advantages, such as non-invasiveness^22^, targeting specificity^23^, high stability^24^, and controllability^25^, thus making NPs an excellent candidate for crossing the BBB to treat brain tumors. Gold, a noble metal, has been used since ancient times. Beyond gold’s currency value, its medicinal potency is also of great interest^26^. To date, various forms of gold nanostructures, such as gold nanospheres, gold nanorods, and gold nanostars, have played crucial roles in diverse aspects of biomedicine, particularly in the field of cancer therapy^27,28^. Generally, gold-based nanomaterials (GBNs) have the advantages of good biocompatibility^29^, low immunogenicity^30^, and high physiological stability^31^. Because the preparation method of GBNs is well developed, their physicochemical properties (i.e., size, shape, and surface charge) can be precisely controlled and adapted to various biomedical applications^32,33^. Moreover, owing to the strong binding affinity of gold nanoparticles (GNPs) to mercaptan, disulfide, and amine, the surfaces of GNPs can be easily modulated with various functional molecules to improve their water dispersity^34^, prolong their blood circulation^35^, and endow them with targeting specificity^36^. In addition to being exceptional drug carriers, GBNs can be simultaneously used as contrast agents and photothermal conversion agents to accurately guide the photothermal ablation of tumor tissues through bioimaging^37,38^. Taken together, GBNs have the potential to serve as an ideal multifunctional carrier across the BBB, for applications in the diagnosis and treatment of brain cancers.

In this review, the state-of-art advances in the development of GBNs for brain cancer treatment are summarized. The effects of physicochemical properties and surface functionalization on therapeutic applications are emphasized, and the most feasible pathways of GBNs penetration of the BBB are speculated upon and discussed. Finally, the challenges and future outlook in the development of GBNs for brain cancer treatment are also described.

## Regulation of the physicochemical properties of GBNs to cross the BBB

Benefiting from their controllable size, shape, and surface charge, GBNs are considered ideal nanocarriers for crossing the BBB and enhancing drug accumulation in brain tumors. In this section, the critical effects of the physicochemical properties of GBNs on their penetration through the BBB are comprehensively reviewed and discussed.

### Size

The particle size-dependent effects on the entry of NPs into cancer cells was considered before all the other properties. In fact, the size-dependent behavior of GBNs in living systems has been widely studied since their first biomedical use. For example, our group has determined the optimal size (50 nm) for passive solid tumor targeting and investigated the size-dependent nucleus-targeting effect systemically^39,40^. Furthermore, we have demonstrated that ultrasmall (~2 nm) GNPs can be applied in nuclear gene delivery without using a nuclear targeting sequence. In general, particles with small sizes (<10 nm) usually have excellent penetration ability, although NPs smaller than 6 nm have been reported to be rapidly metabolized and excreted by the kidney^41,42^. Because size matters in various biological processes, this property should be comprehensively considered in designing carriers to cross the BBB and enter brain tumor tissue.

**Figure 2:**
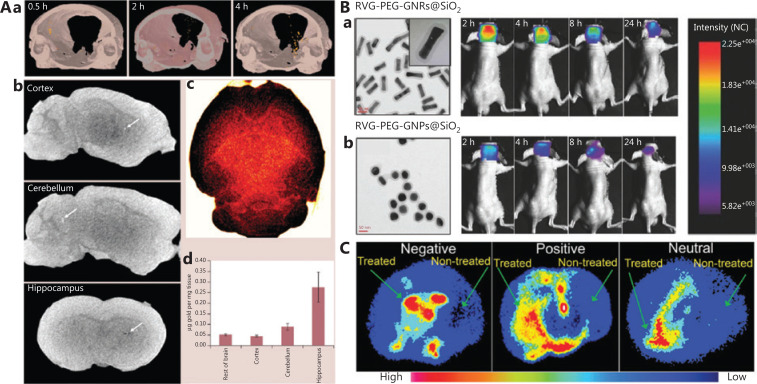
Effects of the physicochemical properties of GBNs on BBB penetration. A. Effect of size on crossing the BBB. (a) Brain CT imaging of 20 nm INS-GNPs, with the yellow dots representing GNPs; CT imaging of 20 nm INS-GNPs in (b) different parts of the brain (white dots indicated by white arrows represent GNPs), and (c) whole brain (gold dots represent GNPs) at 4 h after administration. (d) ICP-MS analysis of GNPs in different brain parts. Reprinted with permission from reference 45. B. Effect of shape on crossing the BBB. TEM images and *in vivo* fluorescence imaging of (a) RVG-PEG-GNRs@SiO_2_ and (b) RVG-PEG-GNPs@SiO_2_. Reprinted with permission from reference 53. C. Effect of surface charge on crossing the BBB. Brain positron emission tomography imaging of GBNs with different charges. The light blue, red, and yellow in the figure indicate the dispersed gold NPs. Reprinted with permission from reference 60.

Owing to their precise size controllability, GNPs are considered an excellent model to explore the size effects on BBB-crossing ability^43,44^. In a previous study, a CCEC model was constructed to study the penetrability of GNPs of the various sizes^45^. Importantly, the authors further quantified the efficiency of insulin-coated gold NPs (INS-GNPs, 20, 50, and 70 nm) *in vivo* by using a Balb/C mouse model (**Figure 2A**). The results revealed that 20 nm insulin-coated GNPs accumulate at higher concentrations in the brain tissue, possibly because of their passage through the gap between CCECs and longer blood circulation. Another study has focused on smaller PEGylated GNPs with core sizes between 4 and 24 nm^46^. By using an *in vitro* transport-permissive brain microvasculature model, the authors demonstrated that larger particle size is negatively correlated with BBB-crossing ability. Similarly, other studies have shown that the smaller the NPs penetrate the BBB more easily^43^. However, despite the more efficient BBB-crossing ability of smaller GNPs, these particles usually show non-negligible toxicity, particularly at sizes below 10 nm^47^. Therefore, the greatest challenge is to improve the biocompatibility of small NPs to achieve efficient and safe BBB penetration.

### Shape

The shape of NPs is regulated to meet the practical needs for functional design^48^. In general, particle shape is closely associated with biological performance, affecting factors such as cellular uptake and bioavailability. For example, nanospheres are endocytosed by cells more rapidly than nanosheets, nanorods, and nanocubes^49^. Nanorods are more easily phagocytosed by macrophages and consequently accumulate to a greater extent than nanorods in tumor tissue. Moreover, the shape can also determine the antimicrobial properties of NPs, for instance, nanostars and nanoflowers have better sterilization effects against *Staphylococcus aureus* than nanospheres^50^. Therefore, investigating the effects of NP shape on crossing the BBB is valuable.

Owing to advances in synthetic methods for nanomaterials in the past few decades, the shape of GNPs can be well controlled^51^. In a cell study, Enea et al.^52^ have demonstrated that gold nanospheres have higher cell uptake efficiency than identically sized gold nanostars in human cerebral microvascular endothelial cells, thus providing a strong basis for further *in vivo* exploration on BBB crossing. Notably, Lee et al.^53^ have prepared a nanoplatform (PEG-RVG-GNRs@SiO_2_) by modifying PEG and peptide (RVG) on the surfaces of mesoporous silicon (SiO_2_) coated gold nanorods (GNRs). PEG-RVG-GNRs@SiO_2_ consistently show higher brain accumulation than the corresponding gold nanosphere-based delivery platform. The enhanced ability of the rod-like NPs to cross the BBB might be ascribable to their curvature being smaller than that of sphere-like NPs, thereby increasing the tendency of nanorods to bind membrane receptors on the surfaces of CCECs (**Figure 2B**). Additionally, shorter GNRs show greater brain accumulation than longer GNRs; this finding may be attributed to long GNRs facing greater resistance from the intercellular fluid or cytoplasmic matrix when crossing the BBB^54^. In short, although determining specific parameters for the geometry of NPs with optimal BBB penetration performance might be difficult, we speculate that NPs with small curvature and appropriate aspect ratios may cross the BBB more efficiently than conventional spherical NPs.

### Surface charge

The surface charge, arising from the ionization of surface functional groups of NPs, can significantly regulate NP cellular uptake, blood circulation half-life, and biodistribution^55^. In general, owing to electrostatic interactions, positively charged NPs are more easily absorbed by cell membranes than negatively or neutrally charged NPs^56^. NPs with negative or neutral charges usually show longer bloodstream circulation times than positively charged NPs, owing to their difficulty in binding to proteins or cells in the blood^57,58^. Additionally, negatively charged NPs have higher liver accumulation than positively charged NPs with similar sizes or shapes, whereas opposite results have been observed in the heart, spleen, and kidney^59^. Therefore, surface charge is another important factor affecting the BBB-crossing ability of NPs.

Recently, Sultan et al.^60^ have used radioisotope-labeled (^64^Cu) gold nanoclusters (GNCs) to verify the effects of surface potential on crossing the BBB. Positron emission tomography imaging has shown that the brain retention of neutrally charged 64Cu-GNCs (−0.04 ± 0.12 mV) is higher at 1 h and 4 h after intravenous injection than that of GNCs with positive (10.8 ± 0.4 mV) and negative (−15.3 ± 4.0 mV) charges. Intriguingly, the brain accumulation of neutrally and negatively charged ^64^Cu-GNCs decreases within 24 h after administration, whereas the uptake of positively charged ^64^Cu-GNCs by brain tumors gradually increases (**Figure 2C**). As a result, positively charged ^64^Cu-GNCs have the highest total accumulation in the brain among the NPs with 3 different surface potentials. This finding should be highly valuable for optimizing the design of nanocarriers to cross the BBB by modulating the surface charge. Similarly, in comparison to negatively charged GNPs (−5.1 mV), ligand-modified positively charged GNPs (+8.6 mV) exhibit ~3.3 folds greater accumulation in the brain^54^. Notably, when the zeta potential of GNPs is as high as +40 mV, only a small number of GNPs accumulate in the brain, thus indicating that the NP surface charge is not proportional to the BBB-crossing ability. This finding may be explained by the excessively high positive charges on the surfaces of the NPs potentially driving more non-specific protein binding, thus accelerating clearance from the blood^61^. Therefore, NPs with a positive surface charge (around +10 mV) can be concluded to the capable of effectively overcoming the BBB and entering the CNS.

Although the controllable synthesis of GBNs with desired physicochemical properties has been well developed, the major obstacle to probing their structural effects on BBB penetration is the inability to control only one physical factor at a time. For example, increasing the size of GNPs usually simultaneously increases their surface charge density, thus preventing single variable studies to evaluate the BBB-crossing ability of GNPs. This situation is even more complicated for the shape effect, because shape switching typically changes both the size and the surface area. Moreover, the surface charges of GNPs can be adjusted by surface functionalization, which in turn changes the hydrodynamic size of the particle. Indeed, we have evaluated the synergistic effects of size and surface charge on cellular uptake^62^ and have found that all cationic GNPs of various sizes enter cells at a significantly higher rate than neutral zwitterionic and anionic GNPs. Interestingly, the cell internalization of neutral and negative NPs decreases with increasing particle size, whereas an opposite tendency is observed for positive NPs. Accordingly, to fully understand effects of NP attributes on their penetration through the BBB, all factors should be considered comprehensively, and mechanisms should be systematically evaluated in greater detail.

## Optimization of the surface functionalization of GBNs to cross the BBB

Once the size or shape is confirmed, NPs usually need specific surface functionalization to meet the requirements of biomedical applications. Importantly, biomolecule conjugation on NP surfaces endows diverse biological functions such as biocompatibility, physiological stability, and targeting specificity^63,64^. In this section, we summarize several common biomolecules for surface modification of GBNs for brain tumor targeting.

### Polymers

To improve the stability of NPs and protect them from agglomeration in some physiological environments, biocompatible surfactants are usually adopted^65^. Owing to spatial resistance and structural specificity, biocompatible polymers have been identified as appropriate candidates to improve the dispersion, decrease clearance by the reticuloendothelial system, and prolong the blood circulation of NPs^66^. Polyethylene glycol (PEG), a biocompatible polymer, is commonly used in the pharmaceutical industry and is also an excellent molecule for the surface functionalization of GBNs. Etame et al.^46^ have synthesized PEGylated GNPs with various PEG chain lengths to explore their ability to penetrate the brain microvasculature. Interestingly, the permeability of GNPs is dramatically improved by PEGylation, and the permeability decreases with increasing PEG molecular weight. As mentioned above, this finding might be due to the increase in particle size after the polymer modification of PEG. In another example, Lu et al.^67^ have performed photoacoustic imaging of brain vasculature in mice by using PEGylated hollow gold nanospheres as a contrast agent. The imaging results clearly showed cerebral vessels with a diameter of approximately 100 µm, thus demonstrating that hollow gold nanospheres are highly enriched in the mouse brain after PEGylation. Beyond PEG, Bishop et al.^68^ have artificially prepared degradable polymer-coated GNPs for the delivery of siRNA and DNA. They have found that the nanosystems are easily internalized by CCECs and distributed in both the cytoplasm and nucleus. Furthermore, poly(2-hydroxypropylmethacrylamide)-coated GNPs has been confirmed to be preferentially taken up by CCECs, as compared with skin endothelial cells^69^. *In vivo* research has demonstrated that polymer-modified GNPs can effectively cross the BBB and accumulate in brain tissue.

In addition to the polymers described above, polymers such as chitosan, poly(lactic-co-glycolic acid) (PLGA), or hyaluronic acid, might also act as surface ligands to enhance the BBB-crossing ability of GBNs to enter brain tumors. For instance, biocompatible GNPs coated with glycol chitosan effectively accumulate in the brain after intravenous administration, mainly because a chitosan-induced increase in adsorption-mediated endocytosis aids in crossing the BBB^70^. Moreover, because PLGA promotes the movement of NPs from endothelial cells to neurons near the BBB and simultaneously decreases the adhesion between particles and the brain parenchyma, the PLGA modification can facilitate the ability of NPs to bypass the BBB and improve their brain tissue permeability^71^. In addition, hyaluronic acid specifically binds CD44 receptors overexpressed on various cancer cells, and it has been widely used for constructing brain tumor-targeting delivery systems^72–74^.

### Proteins

Because of their good biocompatibility, biodegradability, and targeting specificity, proteins have been favored in the construction of drug carriers in recent years^75^. The most representative example is Abraxane, comprising paclitaxel albumin protein-bound particles, which has been approved by the Food and Drug Administration for clinical treatment of breast, non-small cell lung, and pancreatic cancer^76^. The albumin form might substantially affect dispersity and stability, and eventually the functional properties of paclitaxel. Therefore, the protein functionalization choice might have the potential to promote NP penetration of the BBB and enhance the effectiveness of brain cancer treatment.

**Figure 3:**
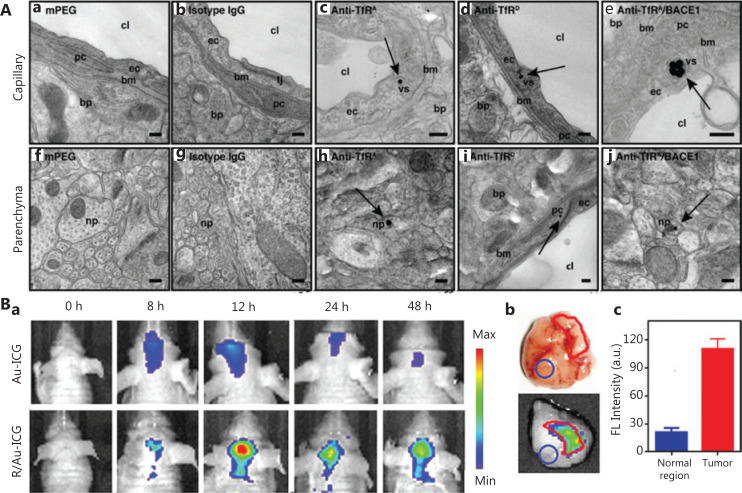
Optimization of the surface functionalization of GBNs to cross the BBB. A. TEM images of GNPs in brain tissue. The black dots indicated by the black arrows represent GNPs. Reprinted with permission from reference 80. B. (a) *In vivo* near-infrared imaging of Au-ICG and R/Au-ICG-treated orthotopic glioma bearing nude mice. (b) *Ex vivo* tumor photograph and fluorescence imaging of intracranial glioma in mice treated with RGD-modified GNPs at 12 h after administration. (c) Corresponding semi-quantitative results of tumors and normal regions. Reprinted with permission from reference 85.

In the basic structure of the BBB, CCECs express specific endocytosis-related receptors on their membranes^77^. Hence, appropriate protein modification of NPs could enable them to cross the BBB *via* receptor-mediated trans-endocytosis. For example, Ruan et al.^78^ have used low-density lipoprotein receptor-associated protein-1 (LRP1) as a specific ligand to modify GNPs (LRP1-PEG-DOX-GNPs). Compared with protein-free NPs, the targeted protein-modified NPs show higher BBB-penetration-efficiency and glioma accumulation without conspicuous adverse effects. Similarly, Cabezon et al.^79^ have modified GNPs with an 8D3 anti-transferrin receptor (anti-TfR) antibody and found that the amount of GNPs in the brain is markedly increased with the surface modification of the protein. In another case, through binding antibody proteins to GNPs, a nanoplatform has been synthesized for targeting transferrin receptors (TfR) on the surfaces of brain capillaries^80^. TfR-targeted GNPs accumulate in the cerebral capillaries and further pass through the BBB into the brain parenchyma, as shown in **Figure 3A**. Importantly, the valency and affinity of antibodies are closely associated with the BBB-penetration-ability of NPs. GNPs modified with high- and low-affinity antibody proteins mediate low and moderate brain uptake, respectively, whereas the introduction of monovalent (bi-specific) antibodies clearly enhances brain uptake of GNPs. Altogether, the multiple highly specific interactions between the modified proteins and membrane receptors can improve the endocytosis of NPs and consequently enhance BBB penetration.

### Peptides

Although peptides are distinguished from proteins by their shorter lengths, they perform equally important biological functions, notably serving as targeting ligands during targeted drug delivery^81^. Commonly used peptides for targeted drug delivery include transcription peptide, RGD peptide, and transferrin peptide. Peptide-modified GNPs have also been demonstrated to enhance the BBB penetration in brain cancer therapy. For example, Zhang et al.^82^ have modified GNPs with glycoprotein-derived peptides from the rabies virus and achieved effective specific biological imaging of nerve cells by taking advantage of the low toxicity and controllable size of GNPs. In a proof-of-application study *in vivo*, Cheng et al.^83^ have reported a 10-fold increase in GNP accumulation in brain tumors after epidermal growth factor receptor peptide modification, in comparison with unmodified GNPs. Furthermore, GNPs with surface modification by the transactivator of transcription peptide can efficiently cross the BBB and deliver therapeutic agents to brain tumor tissues^84^. In a recent publication, an RGD peptide-modified bisulfite-zinc^II^- dipicolylamine-Arg-Gly-Asp [Bis(DPA-Zn)-RGD] has been successfully synthesized for co-assembly with ultrasmall GNPs at brain tumor sites^85^. The nanostructures have been found to overcome both the BBB and the blood-brain tumor barrier (BBTB) for imaging and treatment of *in situ* brain tumors (**Figure 3B**).

Undoubtedly, GNPs with diverse surface functionalization enhance BBB transportation and thus facilitate the treatment of brain cancers. However, the surface functionalization with polymers, proteins, and peptides might simultaneously change the physicochemical properties of the NPs themselves and in turn affect the BBB-crossing ability. Hence, all these factors should be considered and counterbalanced before optimal brain targeting efficiency is achieved.

## Therapeutic strategies for GBNs against brain tumors

Because of the comprehensive evaluation of their physicochemical properties that affect BBB-penetration-ability, GBNs have been applied as carriers of therapeutic agents for brain cancer therapy. Meanwhile, owing to their strong X-ray attenuation ability, high photothermal conversion efficiency, and good resonance Raman scattering characteristics, GBNs also exhibit outstanding performance in computed tomography (CT), photoacoustic, and Raman imaging^86,87^. In the following section, the recent progress in the use of GBNs for drug delivery, bio-imaging, and combination therapy in brain cancer treatment are summarized.

### Therapeutic agent delivery

As mentioned before, chemotherapy is one of the most important methods for the treatment of brain cancer; however, the unique structure of the BBB prevents most therapeutic drugs from entering the brain, thus usually causing treatments to fail. Fortunately, various types of therapeutic molecules can be attached or loaded into GBNs^88^. As a consequence, the solubility and stability of drugs can be improved, and the adverse effects can also be reduced. More importantly, the complexation with GBNs can enhance the transportation of the therapeutic agents into brain tissue.

**Figure 4:**
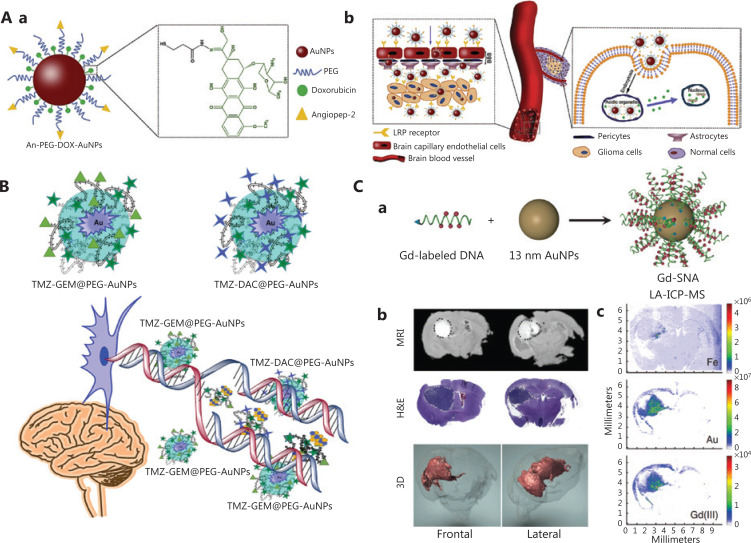
Therapeutic agent delivery *via* GBNs to cross the BBB. A. (a) Illustration of GNPs loaded with DOX. (b) Schematic diagram of DOX-loaded GNPs crossing the BBB to treat glioma. Reprinted with permission from reference 78. B. The interaction between TMZ- gemcitabine@PEG-AuNPs and TMZ-desitabine@PEG-AuNPs for treating brain tumors. Reprinted with permission from reference 91. C. (a) Illustration of Gd (III)-labeled DNA functionalized GNPs. (b) MR imaging of 2 representative coronal slices of the brain after injection of Gd (III)-SNA for 24 h. The upper image shows the location of Gd (III)-SNA in the brain lesions; the middle image is the corresponding H&E stained section; the picture below is the three-dimensional reconstruction of MR images. (c) LA-ICP-MS displays the localization of Fe, Au, and Gd (III) in coronal brain slices of mice after injection of Gd (III)-SNA. Reprinted with permission from reference 93.

The mortality rate associated with glioma, one of the most aggressive and common intracranial tumors, has increased in recent years^89^. To overcome its complex microenvironment and enhance therapeutic efficacy, GBNs are widely used to improve drug delivery to tumor tissue. For example, DOX has been loaded onto the surfaces of GNPs through an acid-responsive hydrazine linker by Ruan et al.^78^, and the GNPs have been further functionalized with angiopep-2, which mediates the BBB penetration of the entire system (**Figure 4A**). Both *in vitro* and *in vivo* results indicate that the gold-based nanosystem has a higher drug delivery efficiency than that of the free drug. The authors then fabricated small GNPs grafted on gelatin NPs and performed surface modification with DOX and Cy5.5; simultaneously, a tandem peptide of RGD and octarginine was functionalized in the system to enhance the BBB-crossing efficiency for treatment of glioma^90^. Because gelatin NPs can be degraded by the matrix metalloproteinases over-expressed at tumor sites, the size of the nanosystem can be reduced from 188.2 nm to 55.9 nm to enhance tumor permeability. The efficiency of drug delivery has been greatly improved by this size-shrinkable transformation. Most recently, Sahli et al.^91^ have demonstrated that temozolomide, gemcitabine, and desitabine can be loaded by hybrid GNPs through electrostatic interactions (**Figure 4B**). The GBNs deliver the drugs more effectively to human U87 malignant glial cells through cell-mediated transport. Notably, this strategy not only distinctly decreases the resistance of glioma cells to temozolomide but also enhances the therapeutic effects on glioma by integrating the synergistic effects of the 3 drugs. Moreover, cisplatin surface engineered GNPs have been found to alter the pharmacokinetics, decrease the systemic toxicity induced by cisplatin, and effectively treat drug-resistant gliomas through synergistic radiosensitization^92^.

In addition to delivering chemotherapy drugs, GBNs have great potential in gene delivery, which can be used to specifically silence target genes in the treatment of brain tumors. In one example, Jensen and coworkers^93^ have preclinically evaluated an RNA interference nanoplatform based on spherical nucleic acid (SNA) gold nanoconjugates, which has been used to regulate oncogene expression in glioblastoma multiforme (**Figure 4C**). After intravenous administration, the gold-based nanoplatform penetrates the BBB and BBTB, achieving knockdown of both the Bcl2-L12 gene (reduced by 26%) and protein (reduced by 40%) in gliomas, and consequently inhibiting tumor growth and extending the survival time of mice. The GNPs coated with Bcl2-L12 specific siRNA (NU-0129) have entered early clinical trials for intravenous injection to treat recurrent glioblastoma^94^. Furthermore, other therapeutic agents such as photosensitizers, antibodies, and probes have been successfully delivered to brain tumors by GBNs^83,95^.

### Bioimaging

Bioimaging is an important visual method to diagnose various diseases, particularly cancers with poor prognosis^96^. GBNs in multimodal imaging not only can track the delivery and accumulation of nanodrugs in real time, but also can distinguish the boundary between normal tissue and tumor tissue, for more accurate and effective guidance of cancer treatment^97^.

**Figure 5:**
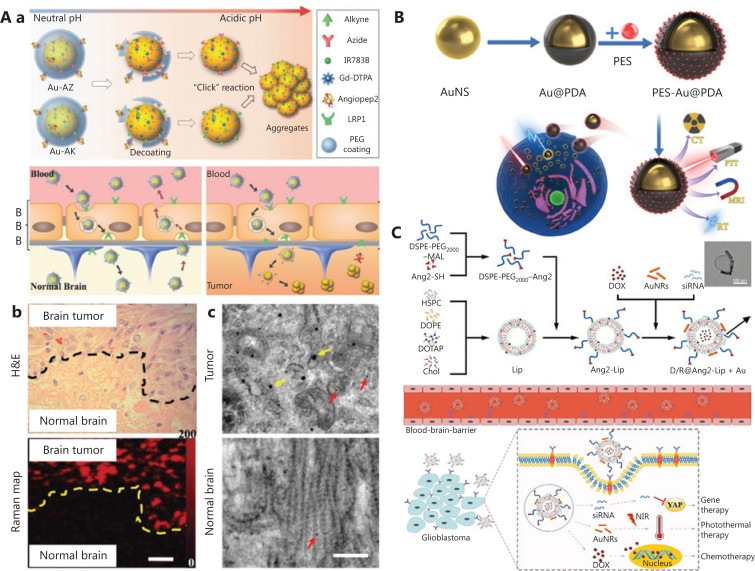
A. (a) Illustration of gold nanoprobes with acid-responsive aggregation to guide brain tumor surgery. (b) The histological H&E stained-photograph and Raman map of the glioma edge area after injection of the Au-AZ/Au-AK mixture for 24 h. (c) TEM images of GNPs in glioma and normal brain at 24 h after injection of Au-AZ/Au-AK. Red arrows indicate monodisperse GNPs, and yellow arrows indicate aggregated GNPs. Reprinted with permission from reference 99. B. Schematic diagram of PES-Au@PDA *via* induction of over-activation of endoplasmic reticulum (ER) stress and a pro-apoptotic UPR cascade to achieve synergistic photothermal therapy and radiotherapy for the treatment of glioblastoma. Reprinted with permission from reference 103. C. Schematic overview of the preparation of D/R@Ang2-Lip+Au and its synergistic chemotherapy, photothermal therapy, and gene therapy to treat glioblastoma. Reprinted with permission from reference 104.

Previous studies have shown that GNPs modified with targeted peptides or proteins can be used to quickly and effectively distinguish glioma cells from non-tumor cells *in vitro* through fluorescence imaging^98^. Typically, GNPs are often used in the design of surgical probes against brain tumors for dual-modal imaging upon a pH stimulus (**Figure 5A**)^99^. Because of the exposure of surface-modified azides and alkyne functional groups of GNPs in acidic conditions, interestingly, the probes can form aggregates *via* click ring addition reactions that activate both magnetic resonance (MR) and surface-enhanced resonance Raman scattering signals. Because the size increases after aggregation, the particles are retained at high levels specifically in the extracellular matrix of the tumor. Furthermore, these gold nanoprobes can penetrate the BBB with the modification of angiopep-2 polypeptide targeting the LRP1 receptor of glioma. Subsequently, brain tumors can be surgically removed under accurate imaging guidance. In another case, a nanostar with multi-modal imaging function has been constructed by using GNPs as the core and the metal-organic framework MIL-88 (Fe) as the shell^100^. Similarly, local enhanced CT/MR/photoacoustic imaging of glioma has provided a basis for accurate and non-invasive diagnosis of glioma *in vivo*.

### Combination therapy

The past few years have seen new trends in clinical oncology from monotherapy to combination therapy to enhance the treatment of cancer by using therapy methods synergistically^101^. For instance, radiotherapy (RT) plays an important role in the treatment of glioma, but radiation tolerance hinders its clinical applications. As mentioned above, GNPs have a high specific surface area and good photothermal conversion efficiency. GNPs have been used as sensitizers for RT to increase X-ray absorption and generate local hyper heat for the thermal ablation of tumors^102^. More recently, GBNs have been used as a radiosensitizer (polydopamine-modified GNPs) and a heat shock protein A5 inhibitor, thus achieving synergistic amplification of RT, photothermal therapy, and pro-apoptotic unfolded protein reactions (**Figure 5B**)^103^. Excitingly, no local tumor recurrence has been observed after 14 d treatment with the multifunctional nanotheranostics in a glioma xenograft nude mouse model. Therefore, the coordinated use of GBNs provides possibilities for solving the tolerance problem of RT in clinical treatment. Furthermore, a combination of brain targeting drug delivery systems and a multi-modal intervention strategy has been developed for glioblastoma therapy. In another recent report, Li et al.^104^ have developed a gold nanorod-angiopeptide-2 modified cationic liposome complex containing DOX and YAP-siRNA (D/R@Ang-2-Lip+Au) (**Figure 5C**). The nanocomposite enables high encapsulation efficiency of chemotherapeutic DOX (95.4%) and genes at an N/P ratio of 20:1. Moreover, it has been shown to have brain glioma targeting ability through a receptor-mediated trans-endocytosis pathway with angiopeptide-2. *In vivo* model studies of *in situ* glioblastoma have indicated that the nanocomposite effectively crosses the BBB and significantly inhibits the growth of glioma, even drug-resistant glioblastoma, through this novel chemotherapy-thermo-gene combination therapy^92,105^. Moreover, GBNs might produce cytotoxic reactive oxygen species under light and ultrasound radiation, thus promoting photodynamic therapy and sonodynamic therapy^106,107^.

## Mechanism of GBNs crossing of the BBB

Although the detailed mechanism through which nanomaterials cross the BBB is not fully understood, the potential pathway could be speculated upon and discussed through systematic study of the GBNs. Generally, there are 2 ways to pass the BBB: passive diffusion and active transport. Passive diffusion is mainly how some small lipophilic molecules (<400 Da) and nutrients, such as CO_2_, O_2_, and alcohol, cross the BBB. Essential nutrients and ions are needed by the brain (e.g., glucose, electrolytes, amino acids, and vitamins), and they bind protein receptors on the cell membrane and form ion channels through the BBB by altering the conformations of membrane proteins^108^. Interestingly, a recent study has shown that ion channels might also play an important role in the transportation of small GNPs across the BBB. After the injection of Ca^2+^, K^+^, and Na^+^ ion channel blockers, the concentrations of GNPs in the mouse brain have been found to decrease by 50%^109^. Notably, the average size of the GNPs used in this study coincides with the size of the ion channels in the range of 0.9–1.5 nm^110^. In this case, we infer that small GNPs can directly cross the BBB through some ion channels. Furthermore, the observation of 50% GNPs brain localization indicates that GNPs might cross the BBB through other pathways. Owing to the high concentrations of extracellular GNPs, some small molecules diffuse from high to low concentration, driven by osmotic pressure. Otherwise, this effect might be due to the dynamic changes in the BBB structure, during which the fluidity of the cell membrane increases, thereby enhancing the penetration of GNPs^111^.

Large GNPs tend to have difficulty in spontaneously penetrating the BBB and instead rely on active transport pathways. As discussed in the above sections, this active transport often requires some surface modification of GNPs, such as with peptides, proteins, or polymers. To date, several specific receptors, such as low-density lipoprotein receptor, insulin receptor, and transferrin receptor, are known to be over-expressed on the front sides of cell membranes of the BBB. For example, GNPs modified with transferrin can specifically bind the transferrin receptors expressed on the blood side of the BBB. After acidification by endocytosis, GNPs are separated from transferrin and released into the brain *via* the BBB^112^. In addition to relying on specific receptors and transporters, GNPs might also be positively electromobilized to cross the BBB through electrostatic interactions with anionic sites on CCECs. This mechanism might also explain why the positively charged GNPs described above penetrate the BBB more easily. Interestingly, researchers have found that some cells, such as mesenchymal stem cells^113^, neutrophils^114^, and red blood cells^115^, can carry substances freely into the brain tissue through the BBB. Hence, exploring the possibility of designing GNPs with enhanced BBB-crossing ability through “riding” cells or membrane functionalization may be a worthy pursuit.

## Conclusions and perspectives

Gold-based nanomaterials provide a powerful reference for the design and development of multifunctional nanoplatforms for the accurate diagnosis and effective treatment of brain cancers. Moreover, the potential of GBNs to cross the BBB also holds great promise for the treatment of some other brain diseases such as Parkinson’s disease^116^, stroke^117^, Alzheimer’s disease^118^, and cerebral vascular embolism^119^. Certainly, each disease has a specific microenvironment, which might cause differences in the physiological barrier. Owing to their controllable physicochemical regulation and tailorable surface modification technology, GBNs might soon be used as a pioneering nanomodel to explore possibilities in treating these brain diseases.

Despite the many favorable properties of GBNs and their great potential in the preclinical treatment of brain tumors, several challenges must be overcome before further translation to clinical settings. The first challenge is biosafety. As discussed before, the size, shape, surface charge, and surface modification of GBNs significantly affect toxicity. These effects are complex and result from multiple factors^120,121^. On the one hand, large spherical GNPs (200–250 nm) accumulate less in the brain, blood, and spleen, whereas the aggregation rate of relatively small NPs (10–15 nm) in these tissues is higher. On the other hand, the tissue accumulation of particles is usually considered an indicator of the toxicity of NPs^43,122,123^. In addition, various surface modifications might result in different bio-effects. Typically, 12 nm GNPs modified with chitosan have a protective effect on brain and liver tissues against lipopolysaccharide-induced tissue toxicity^124^. Active targeting modifications of GNPs might effectively decrease their unnecessary tissue accumulation and consequently their tissue toxicity^125^. Accordingly, the toxicity of GNPs is relative and complex, and their safety assessment also requires systematic examination^126^. However, the effects may be two-sided, such that toxicity may sometimes be beneficial. For example, small GNPs can cause concentration-dependent cell damage but nevertheless can be used to treat brain and neurological bacterial infections^127,128^. Hence, considering the design of GNPs on the basis of one factor alone is insufficient for treating brain tumors; instead, all these factors must be comprehensively considered together to eliminate toxicity.

Although GNPs themselves are biocompatible, non-toxic, and non-immune stimulating materials, the immune stimulation induced by the surface chemical modification of GBNs cannot be ignored^129^. Adjusting the surface modification of 2 nm GNPs has been found to produce different intensities of immune stimulation. GNPs with hydrophobic zwitterionic functionality show the strongest immunological responses, whereas hydrophilic zwitterionic NPs produce a minimal immune response^130^. In contrast, owing to their benefits of immune stimulation, GBNs have been used as an immunotherapy platform for cancer therapy. Clinical studies have shown that, compared with natural tumor necrosis factor (TNF), GNP-TNF conjugates exhibit good immunotherapeutic effects on solid tumors^131^. Moreover, the maximum tolerated dose of TNF conjugated with GNPs for patients is more than 3-fold higher than that of TNF alone, and there are no adverse reactions. Increasing studies demonstrate that combined immunotherapy shows great potential for cancer treatment. That is, the rational design of GBNs to regulate the intensity of immune stimulation may achieve unexpected treatment effects on brain tumors.

The brain tumor environment is more complicated than those of other tumors, owing to the existence of both the BBB and BBTB. These physiological barriers greatly hinder therapeutic agents from effectively exerting their anti-tumor effects, and often trigger tumor resistance. As a result, the limited concentrations of therapeutic agents for brain tumors is another major challenge. This problem could be solved in 2 ways. One is to increase the concentration of the drug, although doing so tends to cause greater biotoxicity^132^. The other is to enhance the targeting efficiency, thereby increasing the drug concentration at particular sites, and decreasing toxic and adverse effects. As mentioned before, optimizing the physicochemical properties and surface functionalization of carriers, such as through positive surface charge or EGF polypeptide modification, might facilitate BBB crossing and thus increase the drug concentrations in brain tissue^57,83^. In terms of drug resistance in brain tumors, some potential drug resistance targets and brain tumor microenvironmental characteristics should be considered. For example, with galactose-oxidized dextran modified GNPs, the modified particles might selectively target TNF-related apoptosis-inducing ligands, thus enhancing the toxicity of DOX to drug-resistant glioma cells over that of free DOX^133^. Collectively, multi-target modifications, design of stimulus-responsive vectors, combination therapies, or a combination of these approaches are worthy strategies for potentially addressing the drug resistance during brain tumor treatment^134^.

Together, although gold-based nanomaterials continue to face challenges in the treatment of brain cancers, we believe that, with rational design, these difficulties could eventually be overcome. We predict more successful and encouraging attempts based on gold nanomaterials for the treatment of brain cancer in the near future, which should provide a valuable reference for constructing more effective nanoplatforms to cure other brain diseases.
